# Calendar

**DOI:** 10.1155/S1463924684000328

**Published:** 1984


					Journal of Automatic Chemistry, Volume 6, Number 3 (July -September 1984), page 163
Calendar
The Automated Analytical Laboratory of
the Future
To be held on 28 November in Sunbury-
on-Thames, UK.
Further information from Dr Clive J.
Jackson, Health and Safety Executive, 403
Edgware Road, London NW2 6LN.
Hewlett-Packard 1985 Analytical
Symposium
To be held from 10 to 14 June 1985 at the
Moat House, Stratford-upon-Avon, UK.
Further information from Mrs Tina
Meats, Hewlett-Packard Ltd, Miller
House, The Ring, Bracknell, Berkshire,
UK.
November 1984
Water for Manufacturing Foods & Drinks
To be held on 19 and 20 November in
London.
Further information from Beverly
Humphrey, Scientific Symposia Ltd, 33-35
Bowling Green Lane, London EC1R ODA.
Annual General Meeting of the Midlands
Region of the Royal Society of
Chemistry's Analytical Division
To be held on 20 November in
Birmingham, UK.
Further information from Miss P. E.
Hutchinson, Royal Society of Chemistry,
Burlington House, London W1 VOBN.
December 1984
Training Requirements for Atomic
Spectroscopy
To be held on 4 December in London.
Further information from Miss P. E.
Hutchinson (above).
Annual General Meeting of the Royal
Society of Chemistry's Analytical
Division's Particle Size Analysis Group/
Pore Size Measurement by Mercury
Porosimetry
To be held on 5 December in London.
Further information from Miss P. E.
Hutchinson (above).
Product Design Assurance in Engineering
To be held from 11 to 13 June 1985 at the
Wembley Conference Centre, London.
Further information from Mrs H. M. W.
Gibbons, Society of Environmental
Engineers, Owles Hall, Buntingford,
Hertfordshire, UK.
IUPAC 1985
To be held from 9 to 13 September 1985 in
Manchester, UK.
Further information from the Royal
Society of Chemistry, Burlington House,
London W1VOBN.
Making the Most of Computers in
Analytical Laboratories
To be held on 21 November in Sheffield,
UK.
Further information from Miss P. E.
Hutchinson (above).
14th Annual Symposium on the Analytical
Chemistry of Pollutants and the 3rd
International Congress on Analytical
Techniques in Environmental Chemistry
To be held from 22-24 November 1984 at
the Palacio de Congresos in Barcelona,
Spain.
Further information from 14th
Annual Symposium--3rd International
Congress--Expoquimia, Avda. Reina Ma.
Christina, Barcelona 4, Spain.
Pharmatech 84 (The Conference for De-
velopment, Formulation, Production and
Quality Assurance and Control Personnel
in the Pharmaceutical Industry)
To be held from 26-29 November at
Fulmer Grange, near Slough, UK.
Further information from Beverly
Humphrey (above).
Annual General Meeting of the Royal
Society of Chemistry's Analytical
Division's Biological Methods Group/
Future Developments in Immunoassay
To be held on 27 November in London.
Further information from Miss P. E.
Hutchinson (above).
Annual General Meeting of the
Electroanalytical Group of the Royal
Society of Chemistry's Analytical
Division/Micro and Microelectronic Solid
State Sensors
To be held on 11 December in London.
Further 'information from Miss P. E.
Hutchinson (above).
Annual General Meeting of the Western
Region of the Royal Society of
Chemistry's Analytical Division/The
Future, if Any, of ASIA (Atomizer Source
ICP Atomic Spectroscopy)
To be held on 14 December in Chepstow,
UK.
Further information from Miss P. E.
Hutchinson (above).
Separation Methods in Clinical Analysis
To be held on 19 December in London.
Further information from Miss P. E.
Hutchinson (above).
1985 MEETINGS
36th Pittsburgh Conference and Expo-
sition on Analytical Chemistry and
Applied Spectroscopy
To be held from 25 February to March
1985 at the Convention Center, New
Orleans, Louisiana, USA.
Further information from David R. Weill
III, Shady Side Academy, 423 Fox Chapel
Road, Pittsburgh, Pennsylvania 15238,
USA.
Colloquium Spectroscopium Internation-
ale XXIV
To be held from 15 to 21 September
1985 in Garmisch-Partenkirchen, FR
Germany.
Further information from CSI XXIV,
Organisationsbi/ro, Institut ffir Spektro-
chemic und angewandte Spektroskopie,
Postfach 778, D4600 Dortmund 1, FR
Germany.
1986 MEETINGS
Analytica 86
To be held from 3 to 6 June 1986 at
Miinchen, FR Germany.
Further information from Miinchener
Messe- und Ausstellungsgesellschaft mbH,
Messegelfinde, Postfaeh 12 10 09, DSO00
Miinchen 12, FR Germany.
SAC 86/3rd BNASS: an International
Conference on Analytical Chemistry
To be held from 20-26 July 1986 at the
University of Bristol, UK.
Further information from The Secretary,
Analytical Division, Royal Society of
Chemistry, Burlington House, London
W1VOBN.
Conference organizers should send full
details of their meeting for inclusion in
'Journal of Automatic Chemistry's'
calendar at least three months before the
event. Information to the Editor, Journal of
Automatic Chemistry, Taylor & Francis
Ltd, 4 John Street, London WC1N 2ET.
163

				

